# Height, mass, and body fat percentage as functions of bmi, gender and age for PSDA from the NHANES 1999-2004 data sets

**DOI:** 10.1186/2046-7648-4-S1-A9

**Published:** 2015-09-14

**Authors:** Arthur Allen, Xu

**Affiliations:** 1 U.S. Coast Guard Office of Search and Rescue, 1 Chelsea Street, New London, CT 06320, USA; 2 U.S. Army Research Institute of Environmental Medicine, Natick, MA 01760, USA

## Introduction

The USARIEM Probability of Survival Decision Aid (PSDA ver 1.0) has gender, height, mass, and body fat percentage inputs [1], but not age, that are either 'User Defined' or categorized as Very High, High, Mean, Low, Very Low. The default values of height and mass are from CDC's National Health and Nutrition Examination Survey (NHANES) [2]; and body fat% are from [3]. However, body fat% is based upon a limited sample size of 665 men and women ages 17 to 65 [3]. The NHANES examines and interviews about 5000 persons each year from representative samples. Each participant is given a unique sequence number and is weighted based upon the portion of the US population that individual represents. The NHANES 1999-2004 data set included the use of Dual-Energy X-ray Absorptiometry (DXA) to measure total body fat % for 11,103 males and 9,687 females age 8 to 85. The total data set had 12,729 males and 13,430 females age 24 to 1019 months (2 -85 years) representing the US population.

## Methods

The NHANES data sets were downloaded from http://www.cdc.gov/nchs/nhanes.htm in SAS XPORT files and converted to Matlab files for analysis and display. The three 2-year data sets of Demographics, Body Measurements, and DXA were combined using the unique sequence numbers, non-respondents were eliminated, and the combined data set was separated by gender and then by age. The weights were combined according to NHANES instructions.

## Results

## Discussion

Analysis of the NHANES 1999-2004 data base was used to update the height, mass, and body fat % in PSDA ver 1.2. Confidence limits were determined to provide results for Very High, High, Low and Very Low estimates of height, mass, and body fat %.

## Conclusion

Using height, mass, body fat% relationships weighted to the entire US population and including the effects of age are more appropriate for the U.S. Coast Guard's use of PSDA than the original PSDA equations for height, mass and body fat % [1].

**Table 1:** Height/Mass on Gender, Age (months) Height (mean) = A + B (Age) + C (Age^2^) and Mass (mean) = D + E(Age) + F (Age^2^)

Gender/Age	A	B	C	D	E	F
Male <= 200 mo	74.866	0.6045	-0.0004281	10.834	0.070687	0.0013087

Male =>200 mo	174.079	0.01441	-1.71739e-05	63.057	0.08842	-7.307256e-05

Female <= 200 mo	65.3569	0.83438	-0.0016530	2.876	0.26357	0.000330611

Female =>200 mo	159.873	0.01678	-1.97931e-05	50.629	0.08937	-7.43095e-05

**Table 2:** Body Fat % on Gender, Age (months), BMI (kg.m^-^^2^) BF% = G + H Age + J [1 -exp(-BMI/K))]

Gender/Age	G	H	J	K
Male <= 240 mo	1.1718	-0.1285	88.2678	29.5664

Male >240 mo	-27.0486	0.0080	72.3643	22.8774

Female <= 240 mo	-9.8860	-0.0211	73.8279	22.0326

Female >240 mo	-37.3765	0.0057	87.5662	14.4364
